# Pharmacological Regulation of Tumor Hypoxia in Model Murine Tumors and Spontaneous Canine Tumors

**DOI:** 10.3390/cancers13071696

**Published:** 2021-04-03

**Authors:** Martin Benej, Jinghai Wu, McKenzie Kreamer, Martin Kery, Sergio Corrales-Guerrero, Ioanna Papandreou, Terence M. Williams, Zihai Li, Edward E. Graves, Laura E. Selmic, Nicholas C. Denko

**Affiliations:** 1 Department of Radiation Oncology, The Ohio State University Wexner Medical Center and OSU Comprehensive Cancer Center, Columbus, OH 43210, USA; martin.benej@osumc.edu (M.B.); jinghai.wu@osumc.edu (J.W.); mckenzie.kreamer@osumc.edu (M.K.); martin.kery@osumc.edu (M.K.); sergio.corralesguerrero@osumc.edu (S.C.-G.); Ioanna.Papandreou@osumc.edu (I.P.); 2 Department of Radiation Oncology, City of Hope National Medical Center, Duarte, CA 91010, USA; terwilliams@coh.org; 3 Pelotonia Institute for Immuno-Oncology, The Ohio State University Comprehensive Cancer Center, Columbus, OH 43210, USA; zihai.li@osumc.edu; 4 Department of Radiation Oncology, Stanford University School of Medicine, Stanford, CA 94305, USA; egraves@stanford.edu; 5 Department of Veterinary Clinical Sciences, College of Veterinary Medicine, The Ohio State University, Columbus, OH 43210, USA; selmic.1@osu.edu

**Keywords:** tumor microenvironment, hypoxia, mitochondria, resistance, papaverine, metabolism

## Abstract

**Simple Summary:**

Tumor hypoxia is a state of low oxygen tension typically occurring in most solid tumors because the oxygen supply does not meet the metabolic demand of the tissue. Hypoxia has been associated with increased resistance to anti-cancer therapy for decades. Reducing oxygen demand with therapeutic targeting of mitochondrial oxidative metabolism can mitigate tumor hypoxia. Here we show that pharmacological regulation of mitochondrial metabolism has a direct impact on the levels of tumor hypoxia in murine tumor models and spontaneous canine soft tissue sarcomas.

**Abstract:**

Background: Hypoxia is found in many solid tumors and is associated with increased disease aggressiveness and resistance to therapy. Reducing oxygen demand by targeting mitochondrial oxidative metabolism is an emerging concept in translational cancer research aimed at reducing hypoxia. We have shown that the U.S. Food and Drug Administration (FDA)-approved drug papaverine and its novel derivative SMV-32 are potent mitochondrial complex I inhibitors. Methods: We used a dynamic in vivo luciferase reporter system, pODD-Luc, to evaluate the impact of pharmacological manipulation of mitochondrial metabolism on the levels of tumor hypoxia in transplanted mouse tumors. We also imaged canine patients with blood oxygen level-dependent (BOLD) MRI at baseline and one hour after a dose of 1 or 2 mg/kg papaverine. Results: We showed that the pharmacological suppression of mitochondrial oxygen consumption (OCR) in tumor-bearing mice increases tumor oxygenation, while the stimulation of mitochondrial OCR decreases tumor oxygenation. In parallel experiments in a small series of spontaneous canine sarcomas treated at The Ohio State University (OSU) Veterinary Medical Center, we observed a significant increase in BOLD signals indicative of an increase in tumor oxygenation of up to 10–50 mm HgO_2_. Conclusion: In both transplanted murine tumors and spontaneous canine tumors we found that decreasing mitochondrial metabolism can decrease tumor hypoxia, potentially offering a therapeutic advantage.

## 1. Introduction

Tumor hypoxia is highly heterogenous and is a function of local oxygen supply and demand within the solid tumor. Hypoxia typically arises through one of two mechanisms. The first is known as diffusion-limited (or chronic) hypoxia, where oxygen diffuses from a blood vessel and is consumed as it passes through cells, dropping in amount the farther it goes and typically reaching stressful levels at 100–150 μm, as originally suggested in [1]. The second mechanism is known as perfusion-limited (or acute) hypoxia, and is caused by inhomogeneity in red blood cell flux through chaotic tumor blood vessels [2,3]. Cellular adaptive response to hypoxia is orchestrated by multiple mechanisms, with the transcription factor hypoxia-inducible factor 1 (HIF-1) playing an important role [4]. The HIF1α subunit is regulated at the protein level by oxygen tension [5]. In well-oxygenated conditions, HIF1α is hydroxylated by prolyl hydroxylases on proline residues 402 and 564. These modified residues are recognized by the von Hippel-Lindau (pVHL) E3 ubiquitin ligase, and HIF1α is marked with polyubiquitin for proteasomal degradation [6]. Because the prolyl hydroxylases use molecular oxygen as a substrate, when oxygen is low, HIF1α is not modified, and the protein becomes stabilized [7]. Several downstream HIF1 target genes function to bring the oxygen supply and demand back in balance, in part by suppressing mitochondrial oxidative metabolism and upregulating the glycolytic flux [8,9]. These complex changes in cancer cell biology promote tumor heterogeneity and are associated with poor clinical prognosis, more aggressive tumor phenotypes, and increased resistance to anti-cancer therapy [10,11,12,13].

Pharmacological manipulation of oxygen demand is an emerging concept in the decades-long (and so far, unfruitful) quest to reduce tumor hypoxia and sensitize tumors to anti-cancer therapy. Mitochondrial oxygen consumption (OCR) constitutes approximately 90% of cellular oxygen demand in oxidative phosphorylation (OXPHOS). This process couples carbon source oxidation with ATP generation in a series of redox reactions transferring electrons from reduced cofactors to molecular oxygen [14]. The electrons are passed through four mitochondrial electron transport chain complexes (I–IV) to oxygen while generating an electrochemical gradient that fuels ATP generation via F0F1 ATP synthase [15]. Mechanistically, a decrease in mitochondrial oxygen consumption would increase the distance of oxygen diffusion within the tumor, allowing chronically hypoxic areas to become re-oxygenated. This principle has been explored in several preclinical strategies supporting the model that mitochondrial OCR has a causal relationship with the levels of tumor hypoxia [16,17,18,19,20].

In this study, we present data showing that biochemical manipulation of mitochondrial OCR modulates the expression of thepODD-Luc transgene in tumors grown in mice. In vitro, cells expressing pODD-Luc showed increased luciferase signals when cultured in hypoxia or with hypoxia mimetics. We also show that mice bearing tumors with pODD-Luc expression had significantly lower level of hypoxic luciferase activity when breathing carbogen (95% O_2_, 5% CO_2_) instead of room air. We further found that strategies that either stabilize HIF1α or stimulate mitochondrial OCR increase pODD-Luc signals, while suppression of OCR decreases pODD-Luc signals. We have previously shown that papaverine, a U.S. Food and Drug Administration (FDA)-approved vasorelaxant with mitochondrial complex I inhibitor activity, effectively reduces hypoxic tumor fractions in vivo [19]. Here we show that its novel papaverine derivative SMV-32, that acts as complex I inhibitor with reduced PDE10A effect, mediates superior OCR inhibition in both orthotopic and heterotopic mouse tumor models. We extended these murine findings in an initial cohort of spontaneous canine soft tissue sarcoma (STS). Patients being treated for spontaneously arising STS at The Ohio State University (OSU) Veterinary Medical Center were recruited to participate in a National Cancer Institute (NCI)-funded test of papaverine as an effective anti-hypoxia agent. We determined changes in blood oxygen level-dependent (BOLD) MRI imaging parameters [21] in these tumors after a single intravenous (I.V.) dose of 1 or 2 mg/kg papaverine. BOLD MRI detects the paramagnetic differences in oxy- versus deoxyhemoglobin [22]. Initial analysis indicated that a fraction of tumors showed significant increases in tumor oxygenation. These findings further establish the direct impact of OCR manipulation on biologically significant tumor hypoxia and support the model that mitochondrial metabolism can drive tumor oxygenation in a potentially therapeutic setting.

## 2. Materials and Methods

### 2.1. Construction, Tumor Growth, and Visualization of pODD-Luc-Expressing Cells

HeLa (cervical cancer, RRID: CVCL_0030) and MiaPaca-2 (MP2) (pancreatic ductal adenocarcinoma, PDAC, RRID: CVCL_0428) cancer cell lines were obtained from American Type Culture Collection (ATCC, Manassas, VA, USA). Stable HeLa pODD and MP2 pODD cell lines were generated by direct transfection with pLenti-pODD-Puro vector generated by subcloning the pODD-Luc cassette from pODD-luciferase-pcDNA3 vector (Addgene #18965, Watertown, MA, USA) into bicistronic pLenti-C-Myc-DDK-IRES-Puro Lentiviral Gene Expression Vector (Origene #PS100069, Rockville, MD, USA). Briefly, the pODD-Luc cassette was amplified using following primers adding AscI and PmeI restriction sites:

ODD-luciferase-AscI-F ATATAggcgcgccgaattcgccaccatggaatt
ODD-luciferase-PmeI-R ATATAgtttaaacattacacggcgatctttccg

The PCR-amplified cassette was then digested with AscI and PmeI restriction enzymes and ligated into pLenti-C-Myc-DDK-IRES-Puro. The correct orientation and sequence were confirmed by Sanger DNA sequencing. HeLa pODD and MP2 pODD transfectants were selected with 2 μg/mL puromycin for 2 weeks. Stable MP2 cells constitutively expressing luciferase reporter gene under cytomegalovirus (CMV) promoter (MP2 CMV) were generated by lentiviral transduction of MP2 cells with pLenti-CMV-Puro-Luc lentiviral vector (Addgene#17477) with 2 μg/mL puromycin selection for 2 weeks. Cells were grown in Dulbecco’s Modified Eagle’s Medium (DMEM) containing 25 mM D-glucose, 4 mM glutamine, and 44 mM sodium bicarbonate in 5% CO_2_, if not stated otherwise. The cells were exposed to hypoxia (1% O_2_) in the H35 Hypoxic Workstation (Hypoxygen) with 5% CO_2_ at 37 °C. In some experiments we used the hypoxia mimetic dimethyloxalyglycine (DMOG), as described. Cell counts and cellular proliferation were established using hemocytometer to determine the cell number and trypan blue exclusion assay to establish the fraction of viable/dead cells. All human cell lines were authenticated using short tandem repeat (STR) profiling within the past 3 years. All experiments were performed with mycoplasma-free cells.

### 2.2. Orthotopic Pancreatic Xenografts

MP2 PDAC cells (1 × 10^6^) or the indicated derivates were implanted in immune-deficient athymic nu/nu mice following Institutional Animal Care and Use Committee (IACUC)-approved protocols. Briefly, an incision was placed in the right flank of anesthetized animals, and the spleen and pancreas were liberated. Cells were mixed 1:1 with Matrigel and implanted in the tail of the pancreas in 10 μL. Incisions were sutured in 2 layers, and the animals were given long-lasting analgesics and allowed to recover from anesthesia. Mice were returned to housing for post-operative care and monitored daily for 4 days. Animals were subsequently checked weekly by palpation to determine tumor growth.

### 2.3. Western Blotting

Proteins were extracted with 1% *v*/*v* Triton X-100, 0.5% *w*/*v* NP-40, 150 mM NaCl, and 50 mM Tris (pH 7.5), quantified using the BCA Kit (Thermo Scientific, Waltham, MA, USA), and separated on 10% SDS-PAGE under reducing conditions. Proteins of interest were detected using antibodies against firefly luciferase (Abcam, Cambridge, MA, USA) and β-actin (Santa Cruz Biotech, Dallas, TX, USA). All replicates of the individual experiments were analyzed from the respective run with appropriate loading controls.

### 2.4. In Vitro Luciferase Assay

Proteins were extracted using Luciferase Assay Lysis Buffer (125 mM Tris-HCl, pH 7.5, 10 mM dithiothreitol (DTT), 10 mM ethylene glycol-bis(β-aminoethyl ether)-N,N,N′,N′-tetraacetic acid (EGTA), 50% glycerol, 5% Triton X-100); 200 μL/sample were transferred to a 96-well plate and D-luciferin was added to the final concentration of 150 μg/mL immediately before the assay. Bioluminescence was measured on a Synergy H1 Hybrid Plate Reader (Biotek, Winooski, VT, USA) after a 10-min incubation period (10 s/sample, gain = 150) in triplicates.

### 2.5. In Vivo Bioluminescent Imaging

All animal experiments were performed according to protocols approved by the OSU institutional IACUC review (protocol #201200000124-R2), with daily veterinarian observation. For heterotopic flank tumor experiments, 1 × 10^6^ HeLa pODD or 5 × 10^6^ MP2 cells were injected subcutaneously (s.c.) into the flanks of 6-week-old female (HeLa pODD) or male (MP2 pODD or MP2 CMV) immunocompromised athymic nu/nu mice. Replicate experiments with groups of 4–6 mice were used for statistical reproducibility. Caliper measurements of opposing diameters were used to calculate the tumor volumes using the modified ellipsoidal formula: tumor volume = ½ (length × width^2^) [23]. Upon reaching 100 mm^3^, the animals were given 100 mg/kg D-luciferin intraperitoneally, and bioluminescence images were captured using the In Vivo Imaging System (IVIS) Lumina II (Perkin Elmer Inc., Waltham, MA, USA). Mice were anesthetized with 1.5% isoflurane, and bioluminescence was measured every 2 min for a period of 25 min to identify the peak response of each mouse to D-luciferin. Bioluminescent data were analyzed using Living Image software (Perkin Elmer Inc.) and calculated as photon flux/s. Obtained individual photon flux peak values were then normalized to baseline for each mouse and averaged per group. Gas inhalation experiments were performed by switching medical air and carbogen gas sources for the IVIS anesthesia vaporizer. Initial baselines were established in medical air under anesthesia, and then the mice were subjected to 30 min of either medical air or carbogen rebreathing in an air-tight chamber while awake. After 30 min, photon flux was established in respective gas sources as a carrier for isoflurane anesthesia.

### 2.6. BOLD MRI Analysis of Canine Tumors

Canine patients with primary sarcomas were enrolled in a trial to evaluate the effects of papaverine delivered at a dose of 1 or 2 mg/kg using blood oxygen level-dependent (BOLD) magnetic resonance imaging (MRI). Dogs were anesthetized with isoflurane in medical air to effect on the bed of a Philips Ingenia 3.0T MR scanner. They were first imaged with T1- (TR: 700 ms, TE: 9 ms, resolution 0.3 × 0.3 × 3 mm) and T2-weighted (TR: 3000 ms, TE: 80 ms, resolution 0.4 × 0.4 × 3 mm) scans in transaxial, coronal, and sagittal orientations to acquire data covering the extent of the tumor. BOLD data were acquired as a series of 60 repeats of a volumetric echo-planar T2-weighted sequence (TR: 3000 ms, TE: 35 ms, resolution 1.6 × 1.6 × 4 mm) collected over a period of 3 min. In addition, a single-slice fast field echo (FFE) sequence (resolution 0.5 × 0.5 × 4 mm) was used to acquire 8 images at echo times from 5 to 75 ms, allowing fitting of pixel T2* values. After these acquisitions, papaverine was delivered intravenously and the BOLD and FFE data were acquired again 1 h post-administration. All data were analyzed using the RT_Image software package. BOLD data were registered using an affine transformation, and the percent signal change was calculated on a per-pixel basis. T2* values were fit on a pixel-by-pixel basis using a Levenberg–Marquardt least squares fit to a monoexponential decay function. A three-dimensional region-of-interest spanning the tumor was defined on the basis of the T2-weighted images and was used to calculate the median change in BOLD signal intensity and T2* over the tumor, as well as the fraction of the tumor that exhibited BOLD signal changes greater than 1, 2, 5, and 10% and that exhibited T2* increases of 1, 2, 5, and 10 ms.

### 2.7. Statistics

Data are presented as mean values ± standard deviation. Student’s *t*-test was used for calculation of significance in differences for pairwise comparisons. In the figures shown, a significance level of *p* ≤ 0.05 is marked with *, *p* ≤ 0.01 with **, and *p* ≤ 0.001 with ***.

## 3. Results

### 3.1. PODD-Luc Signal Increases under Hypoxia In Vitro and In Vivo

**Figure 1:**
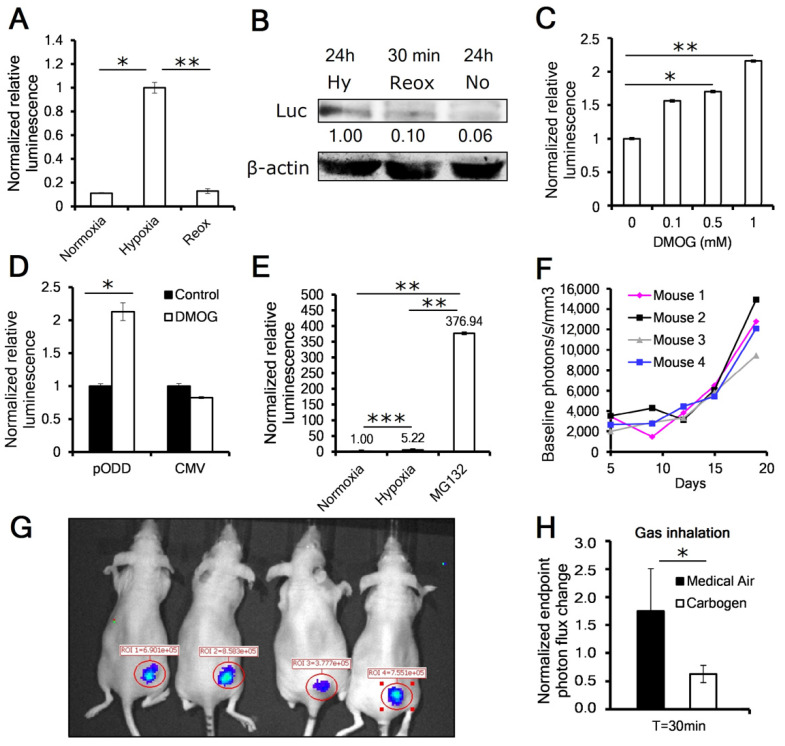
pODD-Luc signal increases under hypoxia in vitro and in vivo. (**A**) Representative in-vitro luciferase assay showing a reporter signal increase in response to 24 h hypoxia and loss of signal after 10 min of reoxygenation in MiaPaca-2 (MP2) pODD cells; values were normalized against hypoxic treatment. *p*-values were calculated against hypoxia by *t*-test (*n* = 3 replicates). (**B**) Western blot of luciferase reporter protein levels in HeLa pODD cells (*n* = 3). (**C**) Dimethyloxalyglycine (DMOG) dose–response analysis in MP2 pODD cell lysates after the indicated treatments (*n* = 3). (**D**) Luciferase assay showing a signal increase in response to 1 mM DMOG in MP2 pODD and no increase in MP2 cells constitutively expressing luciferase gene under cytomegalovirus (CMV) promoter (MP2 CMV). *p*-values were calculated against control by *t*-test (*n* = 3). (**E**) Proteasome inhibition increased MP2 pODD-Luc signals after 1 h of 10 μM MG132 (*n* = 3). (**F**) In vivo luciferase activity of individual HeLa pODD heterotopic flank tumors grown in nu/nu mice with values of photon flux/s normalized to tumor volume (*n* = replicates). (**G**) Representative in vivo imaging assay showing the pODD-Luc signal in HeLa pODD flank tumors (*n* = 4). (**H**) Photon flux in MP2 pODD flank tumors 30 min after animals breathed either medical air or carbogen as indicated (*n* = 4 tumors). Error bars are ±SEM. * *p* < 0.05; ** *p* < 0.01; *** *p* < 0.001.

The pODD-Luc reporter system has previously been used to monitor levels of hypoxia in vitro and in vivo, as was first reported by the Kaelin group [24]. This reporter is a fusion protein connecting the oxygen-dependent degradation domain (ODD) of the HIF1α protein fused to the firefly luciferase reporter. The ODD confers oxygen lability on the luciferase enzyme, which can be stabilized in hypoxia, similarly to the HIF1α protein. We subcloned the original chimeric gene into a bicistronic vector with a puromycin resistance cassette to generate pODD-Luc-Puro. This transgene was introduced into HeLa cervical (HeLa pODD) and MiaPaca-2 pancreatic (MP2 pODD) cancer cell lines and puromycin-resistant pools were selected. We first confirmed the function of the reporter in vitro. We found that the activity of the pODD-Luc reporter cells increased luciferase activity after 24 h in 1% oxygen. We observed a more than 9-fold increase in luminescence and a 10-fold increase in reporter protein when compared to either 21% oxygen conditions or after 10 min of reoxygenation from hypoxic treatment (Figure 1A,B and Figures S1A,B and S2). Likewise, we tested luciferase response in cells treated with dimethyloxalyglycine (DMOG), which inhibits the prolyl hydroxylases (PHDs) that target HIF1α for degradation. We found that 16 h of increasing concentrations of DMOG increased pODD-Luc signals in a dose dependent manner ranging up to 2.2× at 1 mM (Figure 1C).

We confirmed that the increase in the pODD-Luc signal by DMOG was because of reporter stabilization as a result of PHD inhibition by generating MP2 cells expressing constitutive luciferase reporter gene under the CMV promoter (MP2 CMV). These were unresponsive to DMOG treatment (Figure 1D). Because HIF1α is destroyed by proteasomal degradation, we next treated MP2 pODD cells with proteasome inhibitor MG132, and found a dramatic 70-fold increase in pODD-Luc activity when compared to 24 h of hypoxia and a 377-fold induction when compared to normoxia, suggesting that hypoxia stabilizes only a fraction of the expressed reporter protein in vitro (Figure 1E). Finally, to evaluate pODD-Luc reporter activity in vivo, we implanted HeLa pODD into the flanks of immunosuppressed athymic nu/nu mice. We found that the luciferase signal increased as the tumors grew; however, when we normalized for tumor volume, we found that the signal per mm^3^ of tumor also increased, suggesting that as the tumor grew, the amount of hypoxia with it also increased (Figure 1F,G). To confirm that the luciferase signal was responsive to tumor oxygenation, we measured the luciferase signal in MP2 pODD tumor-bearing animals after 30 min of breathing either medical air or carbogen (95% O_2_, 5% CO_2_). As expected, the animals breathing carbogen had approximately one-third the luciferase signal of the animals breathing medical air (Figure 1H).

### 3.2. OCR Manipulation Modulates Levels of Luciferase Activity In Vivo

**Figure 2:**
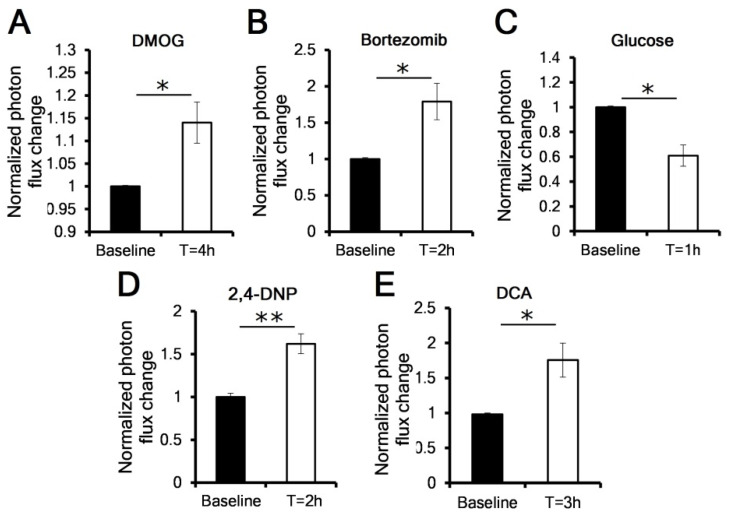
Pharmacological manipulation of pODD-Luc activity in vivo. In vivo bioluminescent image quantification of the pODD-Luc signal in heterotopic flank tumors grown from MP2 ODD cells in immunocompromised mice, after treatment with: (**A**) 4 mg of dimethyloxalyglycine (DMOG) I.P. (*n* = 4 tumors); (**B**) 1 mg/kg of bortezomib I.P. (*n* = 5); (**C**) 2 g/kg of glucose (I.P.) following overnight fasting (*n* = 5); (**D**) 20 mg/kg of 2,4-dinitrophenol (2,4-DNP) I.P., heterotopic tumors (*n* = 6); (**E**) 75 mg/kg of DCA I.P. (*n* = 4). Error bars are ±SEM. *p*-values were calculated against baseline by paired *t*-test. * *p* < 0.05; ** *p* < 0.01. I.P.: intraperitoneally.

Based on our previous findings, our model predicted that biochemical modification of oxygen consumption would affect the overall oxygenation of the tumor [19]. We therefore grew orthotopic pancreatic and heterotopic flank MP2 pODD tumors in immunosuppressed nu/nu mice and monitored relative levels of luciferase activity in real time using the IVIS. To further confirm that the luciferase signal is a reflection of HIF1α protein stability in vivo, we evaluated the impact of inhibiting either the PHDs using DMOG or the proteasome using bortezomib on tumor luciferase activity. As reported for these agents in vitro, in heterotopic MP2 pODD tumor-bearing mice we found that treatment with either a 4 mg dose of DMOG or 1 mg/kg bortezomib delivered intraperitoneally resulted in a significant increase in photon flux at 4- and 2-h post injection, respectively (Figure 2A,B).

As we had seen in vitro, the effect of proteasomal inhibition caused a greater increase in signal than PHD inhibition. The control treatment with vehicle showed no effect on photon flux (Figure S1C). Next, we evaluated the effect of manipulating mitochondrial oxidative metabolism on the dynamic changes of tumor hypoxia. One way to suppress mitochondrial function is to provide cells with a bolus of glucose to produce the Crabtree effect [17], where metabolism shifts from oxidative to glycolytic. Indeed, in mice harboring heterotopic MP2 pODD flank tumors, a 2 g/kg intraperitoneal glucose injection (following an overnight fast) showed a 40% reduction of photon flux at 1 h (Figure 2C). We then investigated whether OCR-stimulating drugs would in turn cause an elevation of hypoxic luciferase activity. Mitochondrial uncouplers such as 2,4-dinitrophenol (2,4-DNP) are protonophoric compounds that dissipate the mitochondrial proton gradient and stimulate a rapid increase of OCR. A single intraperitoneal dose of 20 mg/kg 2,4-DNP produced a significant 1.6-fold increase in photon flux compared to baseline (Figure 2D). Finally, dichloroacetate (DCA) is an inhibitor of pyruvate dehydrogenase kinases 1–4 (PDHKs). Under hypoxic conditions, PDHKs add inhibitory phosphorylations to the pyruvate dehydrogenase PDHA1 subunit to prevent pyruvate oxidation in the tricarboxylic acid (TCA) cycle. Inhibition of PDHKs by DCA has been shown to temporarily increase mitochondrial OCR [8,25]. In a manner similar to 2,4-DNP, intraperitoneal treatment with 75 mg/kg DCA caused a 1.8-fold increase in photon flux, further supporting the model that pharmacological alteration in OCR impacts tumor oxygenation (Figure 2E).

### 3.3. Mitochondrial Complex I Inhibitors Papaverine and SMV-32 Effectively Reduce Hypoxia in Orthotopic Pancreas Cancers

We have previously shown that papaverine (PPV), an FDA-approved vasorelaxant, effectively reduces hypoxia in model breast and lung tumors by previously unrecognized activity inhibiting mitochondrial complex I [19].

**Figure 3:**
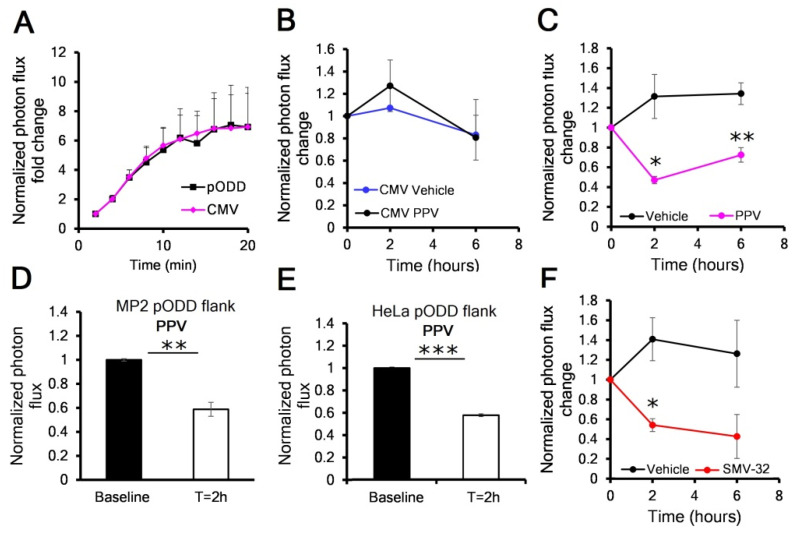
Mitochondrial inhibitors papaverine (PPV) and SMV-32 show reduced pODD-Luc activity (hypoxia) in vivo. (**A**) Acute in vivo imaging pODD-Luc quantification in nu/nu mice bearing orthotopic MP2 pODD or CMV tumors in response to intraperitoneal treatment with D-luciferin (*n* = 3); (**B**) In vivo luciferase activity of MP2 CMV in orthotopic tumors treated with 2 mg/kg PPV or vehicle by tail vein injection (*n* = 4); (**C**) In vivo luciferase activity of MP2 pODD in orthotopic tumors treated with PPV or vehicle by tail vein injection (*n* = 4), with *p*-values calculated against the vehicle; (**D**) In vivo luciferase activity of MP2 pODD in heterotopic flank tumors treated with PPV by tail vein injection (*n* = 5); (**E**) In vivo luciferase activity of Hela pODD-Luc in heterotopic flank tumors treated with PPV by tail vein injection (*n* = 3); (**F**) In vivo luciferase activity of MP2 pODD in orthotopic tumors treated with SMV-32 or vehicle by tail vein injection (*n* = 4), with *p*-values calculated against vehicle. Error bars are ±SEM. Unless otherwise indicated, *p*-values were calculated against baseline by paired *t*-test. * *p* < 0.05; ** *p* < 0.01; *** *p* < 0.001.

Furthermore, we designed novel derivative of PPV, SMV-32, that reduced the vasoactive PDE10A inhibitor activity and enhanced pharmacokinetic parameters. To evaluate the pharmacodynamic properties of PPV and SMV-32 in pancreatic tumors, we grew both orthotopic MP2 pODD and MP2 CMV tumors in immunocompromised nu/nu mice. Both tumor models showed comparable baseline reporter signal in response to D-luciferin (Figure 3A). To rule out non-specific activity of PPV to increase tumor luciferase activity, we tested the oxygen-independent MP2 CMV tumor-bearing mice with a single dose of 2 mg/kg PPV or vehicle by tail vein injection and found no difference in CMV luciferase activity up to 6 h post injection (Figure 3B). When we treated MP2 pODD mice with PPV we observed a 45% reduction in photon flux compared to vehicle (Figure 3C). A similar response was observed in nu/nu mice with flank MP2 pODD and HeLa pODD tumors treated with PPV or SMV-32 (Figure 3D,E and Figure S1F). We also determined the dose and route of delivery effects of PPV on pODD-Luc activity. Intraperitoneal delivery of 2 mg/kg PPV did not reduce the photon flux, suggesting reduced pharmacokinetic properties of intraperitoneal administration. However, a 4 mg/kg dose by tail vein showed greater photon flux reduction than the 2 mg/kg dose, suggesting a dose-dependent response (Figure S1D,E). When we compared 2 mg/kg of each SMV-32 to PPV in orthotopic MP2 pODD tumors, we found that the photon flux of SMV-32-treated mice trended towards a longer duration of action and lower luciferase activity at 6 h when compared to PPV-treated mice (Figure 3 C,F).

### 3.4. Mitochondrial Complex I Inhibitor Papaverine Increases Oxygenation in Spontaneous Canine Sarcomas

**Figure 4:**
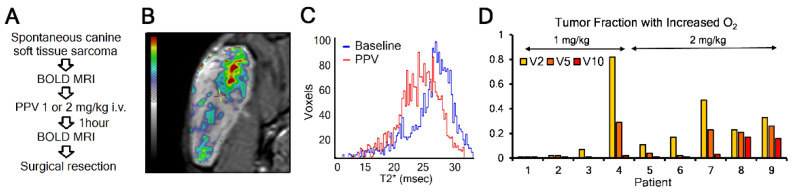
Mitochondrial inhibitor PPV increases oxygenation in spontaneous canine soft tissue sarcoma. (**A**) Schema for imaging patients to determine the effect of PPV on tumor oxygenation. (**B**) Representative overlay image from hip sarcoma in patient 7 with pseudocolor indicating changes of 2 ms (yellow), 5 ms (orange), or 10 ms (red). (**C**) Histogram showing baseline (blue) and post-PPV (red) T2* values for patient 7. (**D**) Waterfall summary of the first 9 patients imaged, showing fractional tumor volumes displaying an increase by either 2, 5, or 10 ms in T2*. The dose of PPV is indicated for each animal.

The murine models of transplanted tumors are genetically simple for both host and tumor genomes. To better-model complex heterogeneous human cancers, we have begun an NCI-funded animal study in client dogs being treated for cancer at the OSU Veterinary Medical Center integrative cancer clinic. This pharmacodynamics study uses blood oxygen level-dependent (BOLD) MRI to determine tumor oxygenation at baseline and one hour after a clinically acceptable dose of 1 or 2 mg/kg PPV delivered intravenously (Figure 4A). When we overlay these images, we can detect the differences in BOLD T2* relaxation signal changes in a voxel-by-voxel analysis. The image in Figure 4B is pseudocolored to show changes from a 0 (transparent) to 10-ms (red) increase in T2* signal. The pattern of changes is reminiscent of the heterogenous pattern of hypoxia that has been reported to exist within a tumor. Histogram analysis of the patient in 4B revealed a clear separation between pre- and post-treatment T2* values (Figure 4C). Figure 4D provides a summary of the first nine evaluable patient images (four treated with 1 mg/kg and five with 2 mg/kg). Because of the heterogeneity seen in these images, we decided to report fractional tumor volumes that increased T2* by either 2 ms, 5 ms, or 10 ms. This level of change is consistent with what has been reported for T2* increases in tumor-bearing mice before and after carbogen breathing, with median changes of only 1–2 ms but with heterogeneous regions of the tumor increasing up to 10 ms [26].

This relative change is consistent with the murine pODD-Luc data presented above, showing a change in signal that is relatively similar between breathing carbogen (Figure 1) and after delivery of 2 mg/kg PPV (Figure 3). Within this heterogeneous response, we found a greater fraction of tumors responding well to the higher dose of 2 mg/kg (3/5) than those responding well to 1 mg/kg (1/4). The signal noise was approximately 1–2 ms, and thus the signal theory would indicate that signals with >2 ms noise would indicate non-random increases in T2*.

## 4. Discussion

Non-invasive analysis of biologically significant hypoxia with ODDluciferase offers a powerful technology to investigate oxygenation in model tumors. In this study, we showed that the ODDluciferase signal is responsive to chemical modification of the HIF1α degradation pathway in vitro and in vivo. These results provide confidence for its use in the analysis of strategies that modify mitochondrial function to change hypoxia. Agents that inhibit mitochondrial function decrease hypoxia and luciferase signals, while agents that stimulate mitochondria increase hypoxia and luciferase signals. This paradigm appears to hold for both homogeneous murine models and heterogenous spontaneous canine tumors where hypoxia has been reported [27,28].

One limitation of these imaging modalities to measure hypoxia is the heterogeneity existing on the cellular level. While it is true that as tumors get larger they show increased hypoxia in the core [29] (Figure 1F), this is an average of many smaller volumes that have a range of oxygenation values. The use of hypoxia marker drugs such as pimonidazole [30] or EF5 [31] has shown that hypoxia can be spread through the tumor in patterns indicative of both diffusion- and perfusion-limited oxygen delivery. These regions can be at the level of tens or hundreds of cells, far too small for imaging technology to detect, but potentially important clinically as these resistant cells can participate in tumor regrowth after treatment [32].

Tumor hypoxia has remained a barrier to effective anti-cancer therapy decades after being recognized as causing radiation resistance [33]. No clinical strategy has been approved by the FDA to reduce hypoxia in order to augment standard therapies. Many attempts have been made to increase oxygen delivery to tumors without clinical success [34]. These failures are partly due to limitations in tumor vessel function. Tumor vessels typically are tortuous, contain breaks and blind ends, and lack a muscular wall. For this reason, even if more oxygen is delivered systemically, the amount that gets to the tumor core is limited. Our alternative approach has been to reduce oxygen demand within the tumor cells. Inhibitors of mitochondrial function appear to be a promising strategy to increase oxygenation in model murine tumors and spontaneous canine soft tissue sarcoma. Our previously published data show that a well-oxygenated normal gastrointestinal (GI) tract is not radiosensitized by papaverine, suggesting that therapeutic gain may be achieved by selectively targeting radiobiological hypoxia in tumors [19]. We have used these preclinical data to support an initial early-phase human clinical trial of papaverine as an adjuvant to radiation therapy that is currently underway at The Ohio State University Comprehensive Cancer Center (NCT03824327). The phase 1 trial is designed to assess the feasibility and tolerability of adding papaverine to stereotactic body radiation therapy in the treatment of patients with non-small cell lung cancer.

## 5. Conclusions

Our findings support the model that drugs that modify mitochondrial metabolism can regulate tumor hypoxia, potentially generating therapeutic gain.

## 6. Patents

A patent has been filed for the use of papaverine or its derivatives as agents to reduce tumor hypoxia by The Ohio State University Innovation Fund. Nicholas Denko is the lead inventor (WO 2020/023537 A1).

## Data Availability

The data presented in this study are available on request from the corresponding author.

## Supplementary Materials

The following are available online at https://www.mdpi.com/article/10.3390/cancers13071696/s1, Figure S1: Additional Data, Figure S2: Uncropped western blot figure.

## References

[1] Thomlinson R.H., Gray L.H. (1955). The histological structure of some human lung cancers and the possible implications for radiotherapy. Br. J. Cancer.

[2] Kimura H., Braun R.D., Ong E.T., Hsu R., Secomb T.W., Papahadjopoulos D., Hong K., Dewhirst M.W. (1996). Fluctuations in red cell flux in tumor microvessels can lead to transient hypoxia and reoxygenation in tumor parenchyma. Cancer Res..

[3] Bader S.B., Dewhirst M.W., Hammond E.M. (2021). Cyclic Hypoxia: An Update on Its Characteristics, Methods to Measure It and Biological Implications in Cancer. Cancers.

[4] Wykoff C.C., Beasley N.J., Watson P.H., Turner K.J., Pastorek J., Sibtain A., Wilson G.D., Turley H., Talks K.L., Maxwell P.H. (2000). Hypoxia-inducible expression of tumor-associated carbonic anhydrases. Cancer Res..

[5] Wang G.L., Semenza G.L. (1993). General involvement of hypoxia-inducible factor 1 in transcriptional response to hypoxia. Proc. Natl. Acad. Sci. USA..

[6] Aprelikova O., Chandramouli G.V., Wood M., Vasselli J.R., Riss J., Maranchie J.K., Linehan W.M., Barrett J.C. (2004). Regulation of HIF prolyl hydroxylases by hypoxia-inducible factors. J. Cell. Biochem..

[7] Wang G.L., Semenza G.L. (1993). Characterization of hypoxia-inducible factor 1 and regulation of DNA binding activity by hypoxia. J. Biol. Chem..

[8] Papandreou I., Cairns R.A., Fontana L., Lim A.L., Denko N.C. (2006). HIF-1 mediates adaptation to hypoxia by actively downregulating mitochondrial oxygen consumption. Cell Metab..

[9] Kim J.W., Tchernyshyov I., Semenza G.L., Dang C.V. (2006). HIF-1-mediated expression of pyruvate dehydrogenase kinase: A metabolic switch required for cellular adaptation to hypoxia. Cell Metab..

[10] Vaupel P. (2008). Hypoxia and aggressive tumor phenotype: Implications for therapy and prognosis. Oncologist.

[11] Gomez C.R. (2016). Editorial: Tumor Hypoxia: Impact in Tumorigenesis, Diagnosis, Prognosis, and Therapeutics. Front. Oncol..

[12] Overgaard J. (2007). Hypoxic radiosensitization: Adored and ignored. J. Clin. Oncol. Off. J. Am. Soc. Clin. Oncol..

[13] Sørensen B.S., Horsman M.R. (2020). Tumor Hypoxia: Impact on Radiation Therapy and Molecular Pathways. Front. Oncol..

[14] Snyder C.M., Chandel N.S. (2009). Mitochondrial regulation of cell survival and death during low-oxygen conditions. Antioxid. Redox Signal..

[15] Mitchell P. (1961). Coupling of Phosphorylation to Electron and Hydrogen Transfer by a Chemi-Osmotic type of Mechanism. Nature.

[16] Zannella V.E., Dal Pra A., Muaddi H., McKee T.D., Stapleton S., Sykes J., Glicksman R., Chaib S., Zamiara P., Milosevic M. (2013). Reprogramming metabolism with metformin improves tumor oxygenation and radiotherapy response. Clin. Cancer Res. Off. J. Am. Assoc. Cancer Res..

[17] Snyder S.A., Lanzen J.L., Braun R.D., Rosner G., Secomb T.W., Biaglow J., Brizel D.M., Dewhirst M.W. (2001). Simultaneous administration of glucose and hyperoxic gas achieves greater improvement in tumor oxygenation than hyperoxic gas alone. Int. J. Radiat. Oncol. Biol. Phys..

[18] Molina J.R., Sun Y., Protopopova M., Gera S., Bandi M., Bristow C., McAfoos T., Morlacchi P., Ackroyd J., Agip A.A. (2018). An inhibitor of oxidative phosphorylation exploits cancer vulnerability. Nat. Med..

[19] Benej M., Hong X., Vibhute S., Scott S., Wu J., Graves E., Le Q.T., Koong A.C., Giaccia A.J., Yu B. (2018). Papaverine and its derivatives radiosensitize solid tumors by inhibiting mitochondrial metabolism. Proc. Natl. Acad. Sci. USA.

[20] Ashton T.M., Fokas E., Kunz-Schughart L.A., Folkes L.K., Anbalagan S., Huether M., Kelly C.J., Pirovano G., Buffa F.M., Hammond E.M. (2016). The anti-malarial atovaquone increases radiosensitivity by alleviating tumour hypoxia. Nat. Commun..

[21] O’Connor J.P.B., Robinson S.P., Waterton J.C. (2019). Imaging tumour hypoxia with oxygen-enhanced MRI and BOLD MRI. Br. J. Radiol..

[22] Padhani A.R., Krohn K.A., Lewis J.S., Alber M. (2007). Imaging oxygenation of human tumours. Eur. Radiol..

[23] Euhus D.M., Hudd C., LaRegina M.C., Johnson F.E. (1986). Tumor measurement in the nude mouse. J. Surg. Oncol..

[24] Safran M., Kim W.Y., O’Connell F., Flippin L., Günzler V., Horner J.W., Depinho R.A., Kaelin W.G. (2006). Mouse model for noninvasive imaging of HIF prolyl hydroxylase activity: Assessment of an oral agent that stimulates erythropoietin production. Proc. Natl. Acad. Sci. USA.

[25] Papandreou I., Goliasova T., Denko N.C. (2011). Anticancer drugs that target metabolism: Is dichloroacetate the new paradigm?. Int. J. Cancer.

[26] Baudelet C., Gallez B. (2002). How does blood oxygen level-dependent (BOLD) contrast correlate with oxygen partial pressure (pO2) inside tumors?. Magn. Reson. Med..

[27] Bruehlmeier M., Kaser-Hotz B., Achermann R., Bley C.R., Wergin M., Schubiger P.A., Ametamey S.M. (2005). Measurement of tumor hypoxia in spontaneous canine sarcomas. Vet. Radiol. Ultrasound Off. J. Am. Coll. Vet. Radiol. Int. Vet. Radiol. Assoc..

[28] Thrall D.E., Rosner G.L., Azuma C., McEntee M.C., Raleigh J.A. (1997). Hypoxia marker labeling in tumor biopsies: Quantification of labeling variation and criteria for biopsy sectioning. Radiother. Oncol. J. Eur. Soc. Ther. Radiol. Oncol..

[29] Klibanov A.L., Hu S. (2019). Monitoring Oxygenation Levels Deep in the Tumor Core: Noninvasive Imaging of Hypoxia, Now in Real-Time 3D. Cancer Res..

[30] Varia M.A., Calkins-Adams D.P., Rinker L.H., Kennedy A.S., Novotny D.B., Fowler W.C., Raleigh J.A. (1998). Pimonidazole: A novel hypoxia marker for complementary study of tumor hypoxia and cell proliferation in cervical carcinoma. Gynecol. Oncol..

[31] Ziemer L.S., Evans S.M., Kachur A.V., Shuman A.L., Cardi C.A., Jenkins W.T., Karp J.S., Alavi A., Dolbier W.R., Koch C.J. (2003). Noninvasive imaging of tumor hypoxia in rats using the 2-nitroimidazole 18F-Efeuropean. J. Nucl. Med. Mol. Imaging.

[32] Brown J.M. (1979). Evidence for acutely hypoxic cells in mouse tumours, and a possible mechanism of reoxygenation. Br. J. Radiol..

[33] Gray L.H., Conger A.D., Ebert M., Hornsey S., Scott O.C. (1953). The concentration of oxygen dissolved in tissues at the time of irradiation as a factor in radiotherapy. Br. J. Radiol..

[34] Torrisi F., Vicario N., Spitale F.M., Cammarata F.P., Minafra L., Salvatorelli L., Russo G., Cuttone G., Valable S., Gulino R. (2020). The Role of Hypoxia and SRC Tyrosine Kinase in Glioblastoma Invasiveness and Radioresistance. Cancers.

