# Supplementary material for: Pharmacological Regulation of Tumor Hypoxia in Model Murine Tumors and Spontaneous Canine Tumors
Source: Cancers (Basel). 2021 Apr 3;13(7):1696. doi: 10.3390/cancers13071696 (PMC8038388; doi:10.3390/cancers13071696)
Supplement: Supplementary file 1 [file cancers-13-01696-s001.pdf]

cancers

MDPI

Supplementary Materials: Pharmacological Regulation of Tumor Hypoxia in Model Murine Tumors and Spontaneous Canine Tumors

Martin Benej, Jinghai Wu, McKenzie Kreamer, Martin Kery, Sergio Corrales-Guerrero, Ioanna Papandreou, Terence M. Williams, Zihai Li, Edward E. Graves, Laura E. Selmic and Nicholas C. Denko

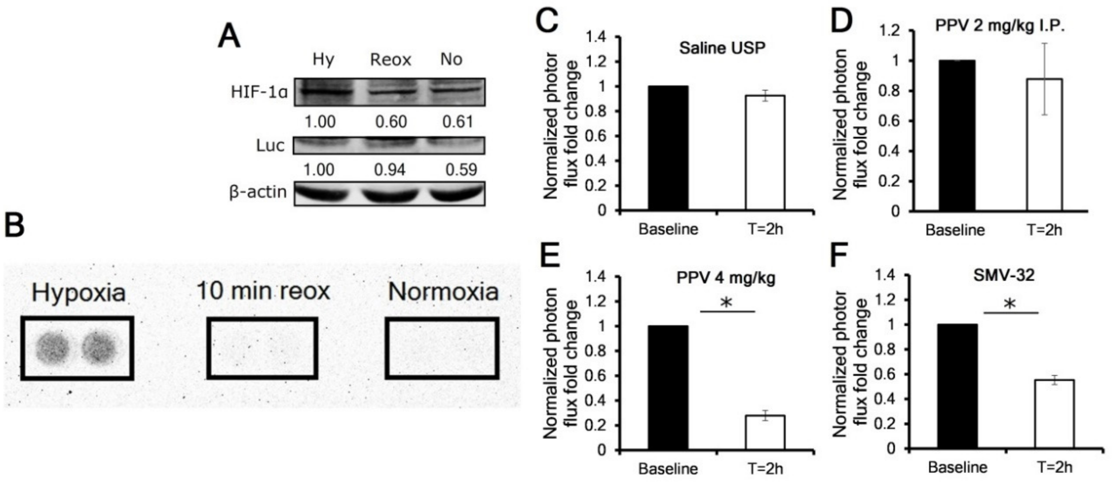

Figure S1. (A,B) Representative western blot of luciferase reporter protein level (**A**) and qualitative bioluminescence image following addition of 150 μg/ml D-luciferin (**B**) in MP2 pODD cells exposed to 24 hours of 1% O_2 with and without 10-minute reoxygenation. **(C–F)** In vivo IVIS imaging quantification of pODD-Luc signal in immunocompromised mice bearing MP2 pODD (**C**,**D**) or HeLa pODD (**E**,**F**) flank tumors, treated with: (**C**) vehicle by tail vein, MP2 pODD tumors, $n = 7$; (**D**) 2 mg/kg PPV intraperitoneally, MP2 pODD tumors, $n = 4$; (**E**) 4 mg/kg PPV by tail vein, HeLa pODD tumors, $n = 3$; and (**F**) 2 mg/kg SMV-32 by tail vein, HeLa pODD, $n = 3$. Error bars are ±SEM. * $p < 0.05$.

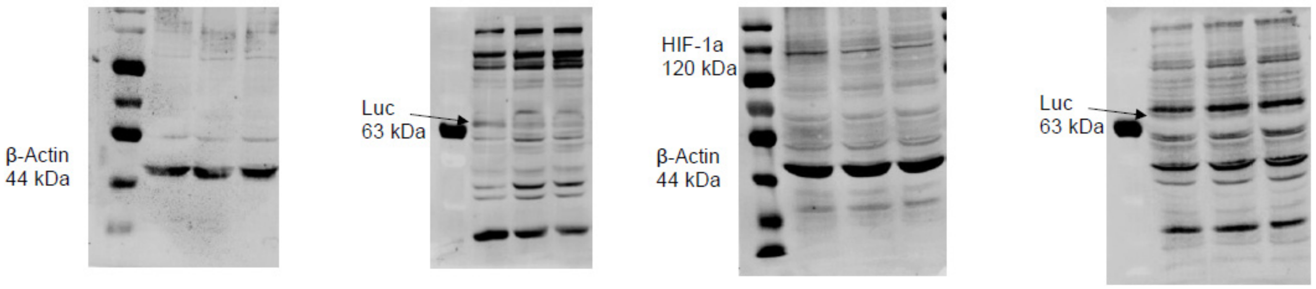

Figure S2. Uncropped western blot figures.
